# Possible Role of PHD Inhibitors as Hypoxia-Mimicking Agents in the Maintenance of Neural Stem Cells’ Self-Renewal Properties

**DOI:** 10.3389/fcell.2018.00169

**Published:** 2018-12-11

**Authors:** Li-Ying Wu, Yun-Ling He, Ling-Ling Zhu

**Affiliations:** ^1^Beijing Institute of Cognition and Brain Sciences, Beijing, China; ^2^State Key Laboratory of Proteomics, Beijing Proteome Research Center, Beijing Institute of Radiation Medicine, Beijing, China; ^3^Co-innovation Center of Neuroregeneration, Nantong University, Nantong, China

**Keywords:** PHDs, inhibitors, NSCs, HIFs, hypoxia

## Abstract

Hypoxia is the most critical factor for maintaining stemness. During embryonic development, neural stem cells (NSCs) reside in hypoxic niches, and different levels of oxygen pressure and time of hypoxia exposure play important roles in the development of NSCs. Such hypoxic niches exist in adult brain tissue, where the neural precursors originate. Hypoxia-inducible factors (HIFs) are key transcription heterodimers consisting of regulatory α-subunits (HIF-1α, HIF-2α, HIF-3α) and a constitutive β-subunit (HIF-β). Regulation of downstream targets determines the fate of NSCs. In turn, the stability of HIFs-α is regulated by prolyl hydroxylases (PHDs), whose activity is principally modulated by PHD substrates like oxygen (O_2_), α-ketoglutarate (α-KG), and the co-factors ascorbate (ASC) and ferrous iron (Fe^2+^). It follows that the transcriptional activity of HIFs is actually determined by the contents of O_2_, α-KG, ASC, and Fe^2+^. In normoxia, HIFs-α are rapidly degraded via the ubiquitin-proteasome pathway, in which PHDs, activated by O_2_, lead to hydroxylation of HIFs-α at residues 402 and 564, followed by recognition by the tumor suppressor protein von Hippel–Lindau (pVHL) as an E3 ligase and ubiquitin labeling. Conversely, in hypoxia, the activity of PHDs is inhibited by low O_2_ levels and HIFs-α can thus be stabilized. Hence, suppression of PHD activity in normoxic conditions, mimicking the effect of hypoxia, might be beneficial for preserving the stemness of NSCs, and it is clinically relevant as a therapeutic approach for enhancing the number of NSCs *in vitro* and for cerebral ischemia injury *in vivo*. This study will review the putative role of PHD inhibitors on the self-renewal of NSCs.

## Introduction

Neural stem cells (NSCs)/neural progenitor cells (NPCs) possess lifelong self-renewal abilities and can differentiate into neurons as well as glial cells such as astrocytes and oligodendrocytes in certain induced conditions (Gage, 2000). Neurogenesis in the adult brain remains controversial. The first evidence of adult hippocampal neurogenesis in the mammalian system was reported 50 years ago (Altman, 1962). However, current opinion is that neurogenesis in the hippocampus decreases rapidly during the first years of life and that neurogenesis in the dentate gyrus does not continue, or is extremely rare in adult humans (Sorrells et al., 2018). In contrast, another view is that human hippocampal neurogenesis from NPCs persists throughout aging (Boldrini et al., 2018). Regardless of this debate, we will review the mechanisms and methods of *in vitro* expansion of NSCs.

Although 21 or 20% of oxygen is considered widely as normoxia in various cell culture methods *in vitro*, physiological normoxia is 2–9% for most cell types (Simon and Keith, 2008). Therefore, hypoxia is actually the microenvironment for most cells, especially stem cells (Mohyeldin et al., 2010). As hypoxia maintains the undifferentiated state of stem cells, including NSCs, hematopoietic stem cells (HSCs), and mesenchymal stem cells (MSCs) (Mazumdar et al., 2010; Mendez-Ferrer et al., 2010; Takubo et al., 2010), the proliferation, quiescence, and fate commitment of stem cells are commonly modulated by hypoxia. Moreover, stem cells have been found to reside mostly in hypoxic niches to sustain a lifelong quiescent or undifferentiated state. Cells respond to hypoxia via hypoxia-inducible factors (HIFs), which are heterodimers composed of a regulatory HIF-α subunit and a constitutive HIF-β subunit (Gordan and Simon, 2007). In general, the effectiveness of hypoxia is mostly achieved through HIF-α. However, HIF-α is extremely unstable under normoxia because it can be degraded by the PHD-mediated ubiquitin-proteasome pathway. Inhibition of PHD activity stabilizes HIF-α and mimics the effects of hypoxia. Therefore, developing PHD inhibitors targeting HIF-α stability would be expected to achieve more specific and effective hypoxia-mimicking effects in normoxia. As mentioned above, in the nervous system, adult NSCs exist in quiescence in limited number (or are even completely absent). Neuronal transplants from NSCs cultured *in vitro* are needed for the treatment of neurodegenerative diseases such as Parkinson’s disease and Alzheimer’s disease as well as conditions like stroke (Goldman, 2016).

In this review, we present recent findings on the roles of hypoxia, HIF transcription factors and PHD inhibitors in NSC proliferation, focusing on the potential application of PHD inhibitors to mimic the effects of hypoxia.

## NSC Self-Renewal/Proliferation Under Hypoxic Conditions

Neural stem cells have the ability to proliferate and differentiate into astrocytes, oligodendrocytes, and neurons (Gage, 2000). Previous studies have shown that in adult mice the main NSC niches are located in the hippocampus and the subventricular zone (SVZ), where they promote stem cell proliferation at low oxygen concentrations (1–5% O_2_) (Mohyeldin et al., 2010). Under anoxic conditions, mitochondrial respiration is inhibited and cellular energy is produced by anaerobic glycolysis, which provides insufficient energy to fully support cell proliferation (Papandreou et al., 2006). Indeed, mild hypoxia (2.5–5% O_2_) is the optimal condition for the proliferation of NSCs in comparison with 1 or 21% O_2_. Santilli et al. (2010) have recently shown that in marked contrast to 2.5–5% O_2_, 1% O_2_ decreased the proliferation of immortalized human NSCs (IhNSCs) and raised the rate of apoptosis. In comparison with IhNSCs, hNSCs were unable to proliferate in 1% O_2_, dying after a few passages, presumably because of cell cycle arrest and inhibition of transcriptional activity (Koshiji et al., 2004; Kaidi et al., 2007; Zhang et al., 2007). Furthermore, Pistollato et al. (2007) found that neural precursor proliferation in the human postnatal brain is enhanced in hypoxic conditions (5% O_2_), while raising oxygen tension to 20% depletes precursors and promotes astrocyte differentiation. Hypoxia-expanded precursors generated 17-fold more oligodendrocytes and when these precursors were expanded in hypoxia and then differentiated in normoxia, oligodendrocyte maturation was further enhanced by 2.5-fold (Pistollato et al., 2007). Hypoxia (5% O_2_) also reduces apoptosis while promoting the proliferation of NSCs, and WNT/β-catenin may be involved in the regulation of NSC proliferation (Cui et al., 2011). Under hypoxia (1.7% O_2_), nuclear orphan receptor TLX acts as a mediator for the proliferation and pluripotency of neural progenitors and is recruited to the *Oct-3/4* (Octamer-binding transcription factor 3/4) proximal promoter, enhancing gene transcription and promoting progenitor proliferation (Chavali et al., 2011). Hypoxia/reoxygenation (H/R) is another method to stimulate NPC proliferation, via activation of the MEK (MAP kinse-ERK kinase)/ERK (extracellular signal-regulated kinase) and the PI3K (phosphoinositide-3-kinase)/AKT signaling pathways through a PKC (protein kinase C)-dependent mechanism. These signals were associated with proliferation of NPCs (Sung et al., 2007).

In addition to the effects on NSC proliferation or self-renewal capacity, hypoxia also increases their survival ability after transplantation into animals with intracerebral hemorrhage. Wakai et al. (2016) have demonstrated that mild hypoxia (5% O_2_, 24 h) enhanced NSC proliferation, upregulated p-AKT via HIF-1α and increased vascular endothelial growth factor (VEGF) production around the wound after transplantation, which favors survival of transplanted cells. Another study indicates that hypoxia regulates the expression of functional metabotropic glutamate receptor (mGluR) in proliferating NSCs and the dynamic expression of mGluRs induced by hypoxia may be one of the mechanisms of hypoxia-stimulated NSC activation (Chen et al., 2016). Interestingly, it has been reported that hypoxic conditioned media from rat cerebral cortical cells promotes NSC proliferation and differentiation into a high percentage of neurons, and these processes may be promoted through PI3K/AKT pathways (Cai et al., 2014).

Hypoxia also has a similar effect on neurogenesis in the animal brain. Our previous study showed that intermittent hypoxia (IH) increased NSC proliferation, newborn neuron survival and migration, spine morphogenesis in the dentate gyrus of the hippocampus, and neurogenesis in the olfactory bulb (Zhu et al., 2005; Zhao et al., 2008). Further evidence suggested that NOTCH1 signaling was activated following IH, which was required for hypoxia-induced neurogenesis (Zhang K. et al., 2014). Ross et al. (2012) reported another hypoxic approach to NSC expansion and neuronal differentiation. An acute (<1 h) *in vivo* exposure to intermittent hypoxia (AIH) was conducted using neonatal C57BL/6 mice, and the isolated NPCs from SVZ tissues grew in neurospheres or adherent monolayers. AIH stimulated the proliferation of NPCs. Therefore, *in vivo* AIH exposure can enhance the viability of SVZ-derived NPC cultures *in vitro* (Ross et al., 2012). Altogether, both *in vivo* and *in vitro* hypoxia stimulation can promote NSC proliferation and a capacity for self-renewal. Since hypoxia has a wide range of effects, revealing the mechanism by which it promotes the proliferation of NSCs will contribute to targeting NSC proliferation *in vitro* and *in vivo*.

## HIF-1 Transcriptional Factor Under Hypoxia

HIF-1α is a regulatory subunit of the HIF-1 transcriptional regulator. The constitutive subunit HIF-1β is present in excess and heterodimerizes with HIF-1α or other bHLH-PAS (basic helix-loop-helix–PER–ARNT–SIM) proteins. Therefore, HIF-1α protein levels determine HIF-1 transcriptional activity (Semenza, 2012). In addition to HIF-1α, HIF-2α was subsequently discovered, which also binds to the common HIF-1β subunit and forms the HIF-2 heterodimer (Tian et al., 1997). While the functions of HIF-1 and HIF-2 are similar, their distribution is different, with HIF-1α being ubiquitously expressed whereas HIF-2α is restricted to certain cell populations and mediates distinct functions (Prabhakar and Semenza, 2012). HIF-3α was originally considered as an inhibitor of HIF-1 via sequestration of HIF-1β. However, some findings indicate that HIF-3 also functions as a transcriptional activator in certain contexts such as in the hypoxic response in zebrafish embryos (Zhang P. et al., 2014). In this article, we focus on the regulation of HIF-1 because it is the most widely studied and represented. In normoxic conditions, prolyl residues 402 and 564 in the HIF-1α oxygen-dependent degradation domain (ODD) are hydroxylated by HIF prolyl hydroxylases (PHDs) (Huang et al., 1998; Semenza, 2001). Subsequently, the hydroxylated HIF-1α is recognized by von Hippel-Lindau protein (VHL), which is responsible for the recruitment of an ubiquitin ligase to target HIF-1α for proteasomal degradation (Ivan et al., 2001). In hypoxic conditions, PHDs are inactivated and the degradation of HIF-1α is blocked. Stabilized HIF-1α constitutes a heterodimer with HIF-1β and directly binds hypoxia-responsive elements (HREs) in the promoter of downstream genes, which are thereby activated (Semenza, 2007; Simon and Keith, 2008). HIF-1 activates the transcription of VEGF and erythropoietin (EPO), which encode angiogenic cytokines or growth factors, leading to angiogenesis and erythropoiesis, and so tissue perfusion improves and O_2_ delivery increases in normal tissues (Semenza, 2013). Another study reported that mice that are homozygous for a null allele at the locus encoding HIF-1α die at embryonic day 10.5 (Yoon et al., 2006). Conditional knockout of HIF-1α in specific cells unveiled its critical roles in hematopoiesis, osteogenesis, and innate immunity (Semenza, 2012), thus highlighting its crucial role in normal development.

## HIF-1 is Associated With Self-Renewal Capacity of NSCs

Hypoxic preconditioning or exposure to sublethal levels of hypoxia enhanced NSC proliferation (Francis and Wei, 2010; Sart et al., 2014). Hypoxic preconditioning (5% O_2_, 24 h) may increase NSC proliferation by acting on the HIF-1α/AKT pathway. Moreover, VEGF as a downstream target of HIF-1α is released in response to hypoxia and promotes cell survival. It is critical for post-stroke cerebral cytoprotection that the transplanted stem cells have the capability to secrete VEGF (Wakai et al., 2016). Hypoxia (0.5% O_2_, 36 h) also promotes the proliferation of neural crest stem cells (NCSCs), which are a population of adult multipotent stem cells through upregulating HIF-1α (Chen et al., 2017). Similarly, intermittent hypoxia (10% O_2_ for 20 cycles, 40-min duration overall) increases NPC populations within the SVZ of the lateral ventricles and in the subgranular zone of the hippocampal dentate gyrus. Further experiments *in vitro* demonstrate that intermittent hypoxia markedly enhances the capacity for expansion of a cultured neurosphere, which is accompanied by increases in a proliferation maker (Ki67), MTT activity, and HIF-1α signaling (Ross et al., 2012).

The underlying mechanisms of HIF-1-mediated NSC proliferation or self-renewal entail the transcriptional expression of VEGF via HIF-1, which in turn stimulates neurogenesis (Harms et al., 2010). Another HIF-1 target gene (EPO) also promotes proliferation of NSCs by modulating NF-kB (nuclear factor-kappa B) signaling (Shingo et al., 2001). Additionally, WNT/β-catenin signaling is implicated in the effects of HIF-1α on NSC proliferation. HIF-1α deletion leads to impaired WNT/β-catenin activity in the hippocampus, with impaired NSC proliferation (Mazumdar et al., 2010; Braunschweig et al., 2015; Hubbi and Semenza, 2015). Of note, our latest unpublished data show that autophagy induced by HIF-1 mediates NSC proliferation. Together, the above studies indicate that HIF promotes NSC self-renewal or proliferation through different signaling pathways. A number of hypoxic conditions are conducive to the self-renewal capacity of NSCs via the HIF-1 pathway. On the other hand, hypoxia does not specifically and exclusively affect NSC proliferation, due to the number of its downstream effectors, apart from the HIF-1 pathway. Furthermore, HIF-1α is unstable under normoxic conditions, and as such is easily degraded by the proteasome system (Prabhakar and Semenza, 2012).

## Hif Ubiquitination Degradation and PHDs

As previously mentioned, in normoxia HIF-1α is subjected to oxygen-dependent hydroxylation by the prolyl hydroxylase domain proteins PHD1-3 at its proline residues 402 and 564, which are followed by the binding of a von Hippel-Lindau protein VHL in an ubiquitin ligase complex (Epstein et al., 2001; Ivan et al., 2001; Jaakkola et al., 2001; Yu et al., 2001). The resulting event is the ubiquitination and proteasomal degradation of HIF-1α. The half-life of HIF-1α is only a few minutes when exposed to 20% O_2_ under standard tissue culture conditions (Prabhakar and Semenza, 2012).

PHDs are recognized as “oxygen sensors” at the cellular level (Kaelin and Ratcliffe, 2008). The PHD family consists of three members, PHD1, PHD2, and PHD3, which share conserved COOH-terminal regions responsible for hydroxylase activity, while at the NH_2_-terminus they are greatly different (Epstein et al., 2001). Each isoform is tissue or cell line-specific as well as subcellular specific (Chen et al., 2015). PHD1 is mainly expressed in the nuclei of the testis and liver (Tambuwala et al., 2010), PHD2 is localized mainly in the cytoplasm in the heart and testis (Selvaraju et al., 2014), and PHD3 has both nuclear and cytoplasmic localization mainly in the heart and liver (Metzen et al., 2003). In addition, they have different regulatory patterns on the modulation of HIF-α: PHD2 is the primary regulator of HIF-1α, and although PHD3 is less active than PHD2 in regulating HIF-1α, it is more likely to target HIF-2α under hypoxic conditions (Appelhoff et al., 2004).

The hydroxylation reaction of HIF-1α is controlled by the activity of PHDs, which is regulated by O_2_, Fe^2+^, α-ketoglutarate, and ascorbic acid (Kaelin and Ratcliffe, 2008; Wong et al., 2013). Under hypoxic conditions, when the hydroxylation reaction is inhibited due to a lack of oxygen, HIF-1α is stabilized and enters the nucleus to form the HIF-1 heterodimer with HIF-1β, which activates the transcription of 100s of target genes and leads to a synergistic adaptation to hypoxia.

It is noteworthy that the prolyl hydroxylase enzymes contain Fe^2+^ in their catalytic centers; therefore, HIF-1α can also be stabilized pharmacologically through the addition of iron chelators such as desferrioxamine (DFO) or iron antagonists such as cobalt chloride (CoCl_2_), in addition to hypoxia. Furthermore, since α-ketoglutarate is a substrate of PHDs, its competitive antagonists [including dimethyloxalylglycine (DMOG)] can stabilize HIF-1α (Epstein et al., 2001). Additionally, multiple proteins have been identified that promote oxygen-dependent degradation of HIF-1α, for example OS9 (ortholog of fungi Yos9p) and RUNX3 (runt related transcription factor 3), which increase the efficiency of hydroxylation of HIF-1α via binding to both HIF-1α and PHD2 (Baek et al., 2005; Lee et al., 2013); and LIMD1 (LIM domains containing 1) alongside RHOBTB3 (Rho-related BTB domain-containing protein 3) can form a complex with HIF-1α, PHD2, and VHL to promote hydroxylation-mediated ubiquitination (Zhang et al., 2015). In any case, PHDs play a crucial role in the instability of HIF-1α. Therefore, inhibition of PHD activity is essential for stabilizing and activating the transcriptional activity of HIF-1.

## PHD Inhibitors and Proliferation of NSCs

As discussed above, the employment of hypoxia-mimetics to provide HIF-1α stabilization might facilitate the expansion of NSCs *in vitro*. However, the commonly used agents for stabilizing HIF-1α, including CoCl_2_, DFO or DMOG, not being specific inhibitors of PHD, have side effects on vital cellular processes. Some agents demonstrate cytotoxicity, spanning from long- (e.g., DFO) to short- (e.g., CoCl_2_) term, to proliferating NPCs, leading to decreased expression of cyclin D1 and nestin in MSCs (Lovejoy and Richardson, 2002; Parmar et al., 2007; Milosevic et al., 2009), while others showed a combination of antiproliferative and cytotoxic effects (e.g., DMOG). However, DMOG-treated human-induced pluripotent stem cell-derived MSCs (hiPSC-MSCs) show improved expressions of HIF-1α and its downstream genes, indicating that DMOG significantly enhances the expression profiles of angiogenic-related factors in hiPSC-MSCs via maintaining HIF-1α stability. In a critical-sized calvarial defect model in rats, DMOG-treated hiPSC-MSCs showed increased angiogenic capacity in the tissue-engineered bone, which results in bone regeneration (Zhang et al., 2016). However, in this study, the side effects of DMOG were not evaluated. A new selective PHD inhibitor FG-4497 has been developed, which stabilizes HIF-1α and induces the expression of HIF-1 target genes. It has been reported that FG-4497 ameliorates mucosal damage in experimental murine colitis (Robinson et al., 2008). In addition, FG-4497 is also reported in HSCs to stabilize HIF-1α protein *in vivo* in the bone marrow (BM), increase the proportion of HSCs and primitive hematopoietic progenitor cells (HPCs) into quiescence, and accelerate blood recovery after severe irradiation (Forristal et al., 2013). Importantly, FG-4497 promotes cell proliferation without being toxic to NPCs and increases the number of cells in normoxia, comparable to hypoxia-induced effects, and is also beneficial for dopaminergic differentiation (Milosevic et al., 2009). Thus, FG-4497 appears to be a non-toxic small molecule, capable of inducing neurogenesis *in vitro*.

Endogenous inhibitors such as reactive oxygen species (ROS) can also block the activity of PHDs, thereby stabilizing HIF-1α. Mitochondria are the main intracellular source of ROS (Solaini et al., 2010; Scherz-Shouval and Elazar, 2011). Other intracellular sources include NADPH oxidases (NOX) (Finkel, 2003). The amount of ROS produced at a cellular level may play a key role in determining NSC fate. In one study, high levels of ROS produced by NOX activity in hypoxic conditions (4% O_2_) were associated with NSC regeneration, while low endogenous ROS levels resulted in a decrease in NSCs’ capacity for self-renewal (Le Belle et al., 2011). The proposed mechanism entailed enhanced ROS-dependent NSC self-renewal and neurogenesis via the PI3K/AKT signaling pathway. Conversely, decreased levels of cellular ROS as a result of pharmacological or genetic manipulations were shown to interfere with NSC function *in vitro* and *in vivo* (Kim and Wong, 2009; Chuikov et al., 2010).

**FIGURE 1:**
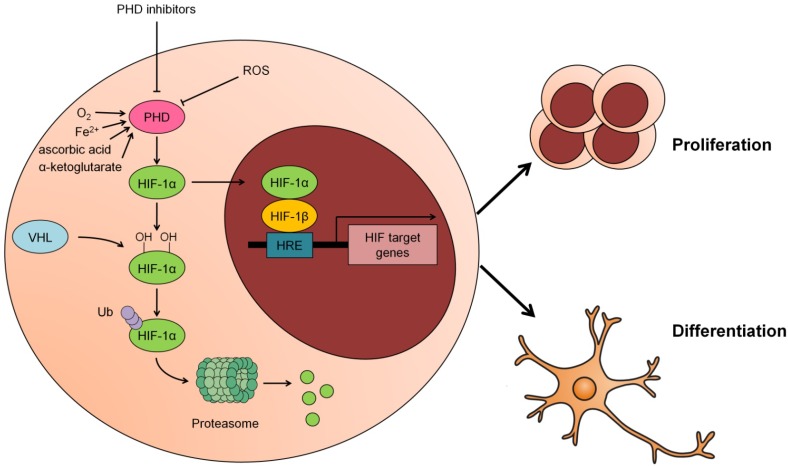
Modulation of PHD-HIF1 signaling on neural stem cells (NSCs). Under normoxia, HIF-1α is hydroxylated by PHD at 402 and 564 residues and subsequently recognized and ubiquitinated by the E3 ligase VHL, hereafter, degraded in the proteasome. In response to hypoxia, ROS or PHD inhibitors, the activity of PHD is suppressed and the hydroxylation of HIF-1α by PHD is inhibited. As a result, the stabilized HIF-1α translocates to the nucleus and partners with HIF-1β to form a heterodimer that transcriptionally activates its downstream target genes to promote proliferation or differentiation of NSCs.

PHD activity is closely related to ROS levels in many systems. Relatively low levels of ROS increase PHD activity, whereas high ROS levels stabilize HIF-1α as a result of inactivating PHDs (Callapina et al., 2005; Fedotcheva et al., 2006; Hamanaka and Chandel, 2009). However, to date, the mechanism by which ROS directly affect PHD function has not been clarified, since stabilization of HIF-1α can occur independently of mitochondrial ROS production during hypoxia, indicating that PHD activity may not be directly regulated by ROS (Wong et al., 2013). One study proposed that H_2_O_2_ may affect PHD activity more directly through a local Fenton reaction (Gerald et al., 2004). Although ROS have been implicated in the regulation of PHD activity, this modulation in NSCs has not been reported. The graphical abstract reported in Figure 1 summarizes these studies and puts forward the concept that the proliferation and differentiation of NSCs are regulated by upstream PHD activity and the core transcription factor HIF-1.

## Conclusion and Future Perspectives

As previously described, hypoxia-dependent regulation of NSC proliferation can be simulated with pharmacological interventions acting via HIF-1α stabilization. Several PHD inhibitors have the potential to promote NSC proliferation; however, their side effects hinder their use. Therefore, the development of new specific and effective PHD inhibitors may offer a valid tool for the application of NSC transplantation in neurodegenerative diseases. Additionally, targeted hypoxia-mimicking agents need to be developed specifically for neural cells for future clinical application.

## Author Contributions

L-YW and L-LZ discussed and wrote the manuscript. L-YW and Y-LH approved the final version to be published.

## Conflict of Interest Statement

The authors declare that the research was conducted in the absence of any commercial or financial relationships that could be construed as a potential conflict of interest.

## References

[B1] Altman J. (1962). Are new neurons formed in the brains of adult mammals? *Science* 135 1127–1128. 10.1126/science.135.3509.1127 13860748

[B2] Appelhoff R. J. Tian Y. M. Raval R. R. Turley H. Harris A. L. Pugh C. W. (2004). Differential function of the prolyl hydroxylases PHD1, PHD2, and PHD3 in the regulation of hypoxia-inducible factor. *J. Biol. Chem.* 279 38458–38465. 10.1074/jbc.M406026200 15247232

[B3] Baek J. H. Mahon P. C. Oh J. Kelly B. Krishnamachary B. Pearson M. (2005). OS-9 interacts with hypoxia-inducible factor 1alpha and prolyl hydroxylases to promote oxygen-dependent degradation of HIF-1alpha. *Mol. Cell* 17 503–512. 10.1016/j.molcel.2005.01.011 15721254

[B4] Boldrini M. Fulmore C. A. Tartt A. N. Simeon L. R. Pavlova I. Poposka V. (2018). Human hippocampal neurogenesis Persists throughout Aging. *Cell Stem Cell* 22 589.e–599.e. 10.1016/j.stem.2018.03.015 29625071 PMC5957089

[B5] Braunschweig L. Meyer A. K. Wagenfuhr L. Storch A. (2015). Oxygen regulates proliferation of neural stem cells through Wnt/beta-catenin signalling. *Mol. Cell. Neurosci.* 67 84–92. 10.1016/j.mcn.2015.06.006 26079803

[B6] Cai M. Zhou Y. Zhou B. Lou S. (2014). Hypoxic conditioned medium from rat cerebral cortical cells enhances the proliferation and differentiation of neural stem cells mainly through PI3-K/Akt pathways. *PLoS One* 9:e111938. 10.1371/journal.pone.0111938 25386685 PMC4227679

[B7] Callapina M. Zhou J. Schmid T. Kohl R. Brune B. (2005). NO restores HIF-1alpha hydroxylation during hypoxia: role of reactive oxygen species. *Free Radic. Biol. Med.* 39 925–936. 10.1016/j.freeradbiomed.2005.05.009 16140212

[B8] Chavali P. L. Saini R. K. Matsumoto Y. Agren H. Funa K. (2011). Nuclear orphan receptor TLX induces Oct-3/4 for the survival and maintenance of adult hippocampal progenitors upon hypoxia. *J. Biol. Chem.* 286 9393–9404. 10.1074/jbc.M110.167445 21135096 PMC3059011

[B9] Chen C. C. Hsia C. W. Ho C. W. Liang C. M. Chen C. M. Huang K. L. (2017). Hypoxia and hyperoxia differentially control proliferation of rat neural crest stem cells via distinct regulatory pathways of the HIF1alpha-CXCR4 and TP53-TPM1 proteins. *Dev. Dyn.* 246 162–185. 10.1002/dvdy.24481 28002632

[B10] Chen X. Liu Y. Zhang Z. Miao X. Tian Y. Yao L. (2016). Hypoxia stimulates the proliferation of rat neural stem cells by regulating the expression of metabotropic glutamate receptors: an in vitro study. *Cell. Mol. Biol.* 62 105–114. 10.14715/cmb/2016.62.3.18 27064882

[B11] Chen Y. Zhang H. S. Fong G. H. Xi Q. L. Wu G. H. Bai C. G. (2015). PHD3 stabilizes the tight junction protein occludin and protects intestinal epithelial barrier function. *J. Biol. Chem.* 290 20580–20589. 10.1074/jbc.M115.653584 26124271 PMC4536461

[B12] Chuikov S. Levi B. P. Smith M. L. Morrison S. J. (2010). Prdm16 promotes stem cell maintenance in multiple tissues, partly by regulating oxidative stress. *Nat. Cell Biol.* 12 999–1006. 10.1038/ncb2101 20835244 PMC2948585

[B13] Cui X. P. Xing Y. Chen J. M. Dong S. W. Ying D. J. Yew D. T. (2011). Wnt/beta-catenin is involved in the proliferation of hippocampal neural stem cells induced by hypoxia. *Ir. J. Med. Sci.* 180 387–393. 10.1007/s11845-010-0566-3 20811817

[B14] Epstein A. C. Gleadle J. M. McNeill L. A. Hewitson K. S. O’Rourke J. Mole D. R. (2001). *C. elegans* EGL-9 and mammalian homologs define a family of dioxygenases that regulate HIF by prolyl hydroxylation. *Cell* 107 43–54. 10.1016/S0092-8674(01)00507-4 11595184

[B15] Fedotcheva N. I. Sokolov A. P. Kondrashova M. N. (2006). Nonezymatic formation of succinate in mitochondria under oxidative stress. *Free Radic. Biol. Med.* 41 56–64. 10.1016/j.freeradbiomed.2006.02.012 16781453

[B16] Finkel T. (2003). Oxidant signals and oxidative stress. *Curr. Opin. Cell Biol.* 15 247–254. 10.1016/S0955-0674(03)00002-4 12648682

[B17] Forristal C. E. Winkler I. G. Nowlan B. Barbier V. Walkinshaw G. Levesque J. P. (2013). Pharmacologic stabilization of HIF-1alpha increases hematopoietic stem cell quiescence in vivo and accelerates blood recovery after severe irradiation. *Blood* 121 759–769. 10.1182/blood-2012-02-408419 23243286

[B18] Francis K. R. Wei L. (2010). Human embryonic stem cell neural differentiation and enhanced cell survival promoted by hypoxic preconditioning. *Cell Death Dis.* 1 e22. 10.1038/cddis.2009.22 21364630 PMC3032335

[B19] Gage F. H. (2000). Mammalian neural stem cells. *Science* 287 1433–1438. 10.1126/science.287.5457.1433 10688783

[B20] Gerald D. Berra E. Frapart Y. M. Chan D. A. Giaccia A. J. Mansuy D. (2004). JunD reduces tumor angiogenesis by protecting cells from oxidative stress. *Cell* 118 781–794. 10.1016/j.cell.2004.08.025 15369676

[B21] Goldman S. A. (2016). Stem and progenitor cell-based therapy of the central nervous system: hopes, hype, and wishful thinking. *Cell Stem Cell* 18 174–188. 10.1016/j.stem.2016.01.012 26849304 PMC5310249

[B22] Gordan J. D. Simon M. C. (2007). Hypoxia-inducible factors: central regulators of the tumor phenotype. *Curr. Opin. Genet. Dev.* 17 71–77. 10.1016/j.gde.2006.12.006 17208433 PMC3215290

[B23] Hamanaka R. B. Chandel N. S. (2009). Mitochondrial reactive oxygen species regulate hypoxic signaling. *Curr. Opin. Cell Biol.* 21 894–899. 10.1016/j.ceb.2009.08.005 19781926 PMC2787901

[B24] Harms K. M. Li L. Cunningham L. A. (2010). Murine neural stem/progenitor cells protect neurons against ischemia by HIF-1alpha-regulated VEGF signaling. *PLoS One* 5:e9767. 10.1371/journal.pone.0009767 20339541 PMC2842303

[B25] Huang L. E. Gu J. Schau M. Bunn H. F. (1998). Regulation of hypoxia-inducible factor 1alpha is mediated by an O2-dependent degradation domain via the ubiquitin-proteasome pathway. *Proc. Natl. Acad. Sci. U.S.A.* 95 7987–7992. 10.1073/pnas.95.14.7987 9653127 PMC20916

[B26] Hubbi M. E. Semenza G. L. (2015). Regulation of cell proliferation by hypoxia-inducible factors. *Am. J. Physiol. Cell Physiol.* 309 C775–C782. 10.1152/ajpcell.00279.2015 26491052 PMC4683214

[B27] Ivan M. Kondo K. Yang H. Kim W. Valiando J. Ohh M. (2001). HIFalpha targeted for VHL-mediated destruction by proline hydroxylation: implications for O2 sensing. *Science* 292 464–468. 10.1126/science.1059817 11292862

[B28] Jaakkola P. Mole D. R. Tian Y. M. Wilson M. I. Gielbert J. Gaskell S. J. (2001). Targeting of HIF-alpha to the von Hippel-Lindau ubiquitylation complex by O2-regulated prolyl hydroxylation. *Science* 292 468–472. 10.1126/science.1059796 11292861

[B29] Kaelin W. G. Jr. Ratcliffe P. J. (2008). Oxygen sensing by metazoans: the central role of the HIF hydroxylase pathway. *Mol. Cell* 30 393–402. 10.1016/j.molcel.2008.04.009 18498744

[B30] Kaidi A. Williams A. C. Paraskeva C. (2007). Interaction between beta-catenin and HIF-1 promotes cellular adaptation to hypoxia. *Nat. Cell Biol.* 9 210–217. 10.1038/ncb1534 17220880

[B31] Kim J. Wong P. K. (2009). Loss of ATM impairs proliferation of neural stem cells through oxidative stress-mediated p38 MAPK signaling. *Stem Cells* 27 1987–1998. 10.1002/stem.125 19544430

[B32] Koshiji M. Kageyama Y. Pete E. A. Horikawa I. Barrett J. C. Huang L. E. (2004). HIF-1alpha induces cell cycle arrest by functionally counteracting Myc. *EMBO J.* 23 1949–1956. 10.1038/sj.emboj.7600196 15071503 PMC404317

[B33] Le Belle J. E. Orozco N. M. Paucar A. A. Saxe J. P. Mottahedeh J. Pyle A. D. (2011). Proliferative neural stem cells have high endogenous ROS levels that regulate self-renewal and neurogenesis in a PI3K/Akt-dependant manner. *Cell Stem Cell* 8 59–71. 10.1016/j.stem.2010.11.028 21211782 PMC3018289

[B34] Lee S. H. Bae S. C. Kim K. W. Lee Y. M. (2013). RUNX3 inhibits hypoxia-inducible factor-1α protein stability by interacting with prolyl hydroxylases in gastric cancer cells. *Oncogene* 33:1458. 10.1038/onc.2013.76 23542169

[B35] Lovejoy D. B. Richardson D. R. (2002). Novel “hybrid” iron chelators derived from aroylhydrazones and thiosemicarbazones demonstrate selective antiproliferative activity against tumor cells. *Blood* 100 666–676. 10.1182/blood.V100.2.666 12091363

[B36] Mazumdar J. O’Brien W. T. Johnson R. S. LaManna J. C. Chavez J. C. Klein P. S. (2010). O2 regulates stem cells through Wnt/beta-catenin signalling. *Nat. Cell Biol.* 12 1007–1013. 10.1038/ncb2102 20852629 PMC3144143

[B37] Mendez-Ferrer S. Michurina T. V. Ferraro F. Mazloom A. R. Macarthur B. D. Lira S. A. (2010). Mesenchymal and haematopoietic stem cells form a unique bone marrow niche. *Nature* 466 829–834. 10.1038/nature09262 20703299 PMC3146551

[B38] Metzen E. Berchner-Pfannschmidt U. Stengel P. Marxsen J. H. Stolze I. Klinger M. (2003). Intracellular localisation of human HIF-1 alpha hydroxylases: implications for oxygen sensing. *J. Cell Sci.* 116 1319–1326. 10.1242/jcs.00318 12615973

[B39] Milosevic J. Adler I. Manaenko A. Schwarz S. C. Walkinshaw G. Arend M. (2009). Non-hypoxic stabilization of hypoxia-inducible factor alpha (HIF-alpha): relevance in neural progenitor/stem cells. *Neurotoxicol. Res.* 15 367–380. 10.1007/s12640-009-9043-z 19384570

[B40] Mohyeldin A. Garzon-Muvdi T. Quinones-Hinojosa A. (2010). Oxygen in stem cell biology: a critical component of the stem cell niche. *Cell Stem Cell* 7 150–161. 10.1016/j.stem.2010.07.007 20682444

[B41] Papandreou I. Cairns R. A. Fontana L. Lim A. L. Denko N. C. (2006). HIF-1 mediates adaptation to hypoxia by actively downregulating mitochondrial oxygen consumption. *Cell Metab.* 3 187–197. 10.1016/j.cmet.2006.01.012 16517406

[B42] Parmar K. Mauch P. Vergilio J. A. Sackstein R. Down J. D. (2007). Distribution of hematopoietic stem cells in the bone marrow according to regional hypoxia. *Proc. Natl. Acad. Sci. U.S.A.* 104 5431–5436. 10.1073/pnas.0701152104 17374716 PMC1838452

[B43] Pistollato F. Chen H. L. Schwartz P. H. Basso G. Panchision D. M. (2007). Oxygen tension controls the expansion of human CNS precursors and the generation of astrocytes and oligodendrocytes. *Mol. Cell. Neurosci.* 35 424–435. 10.1016/j.mcn.2007.04.003 17498968

[B44] Prabhakar N. R. Semenza G. L. (2012). Adaptive and maladaptive cardiorespiratory responses to continuous and intermittent hypoxia mediated by hypoxia-inducible factors 1 and 2. *Physiol. Rev.* 92 967–1003. 10.1152/physrev.00030.2011 22811423 PMC3893888

[B45] Robinson A. Keely S. Karhausen J. Gerich M. E. Furuta G. T. Colgan S. P. (2008). Mucosal protection by hypoxia-inducible factor prolyl hydroxylase inhibition. *Gastroenterology* 134 145–155. 10.1053/j.gastro.2007.09.033 18166352 PMC2194638

[B46] Ross H. H. Sandhu M. S. Cheung T. F. Fitzpatrick G. M. Sher W. J. Tiemeier A. J. (2012). In vivo intermittent hypoxia elicits enhanced expansion and neuronal differentiation in cultured neural progenitors. *Exp. Neurol.* 235 238–245. 10.1016/j.expneurol.2012.01.027 22366327 PMC4089987

[B47] Santilli G. Lamorte G. Carlessi L. Ferrari D. Rota Nodari L. Binda E. (2010). Mild hypoxia enhances proliferation and multipotency of human neural stem cells. *PLoS One* 5:e8575. 10.1371/journal.pone.0008575 20052410 PMC2797394

[B48] Sart S. Ma T. Li Y. (2014). Preconditioning stem cells for in vivo delivery. *Biores. Open Access* 3 137–149. 10.1089/biores.2014.0012 25126478 PMC4120806

[B49] Scherz-Shouval R. Elazar Z. (2011). Regulation of autophagy by ROS: physiology and pathology. *Trends Biochem. Sci.* 36 30–38. 10.1016/j.tibs.2010.07.007 20728362

[B50] Selvaraju V. Parinandi N. L. Adluri R. S. Goldman J. W. Hussain N. Sanchez J. A. (2014). Molecular mechanisms of action and therapeutic uses of pharmacological inhibitors of HIF-prolyl 4-hydroxylases for treatment of ischemic diseases. *Antioxid. Redox Signal.* 20 2631–2665. 10.1089/ars.2013.5186 23992027 PMC4026215

[B51] Semenza G. L. (2001). HIF-1, O(2), and the 3 PHDs: how animal cells signal hypoxia to the nucleus. *Cell* 107 1–3. 10.1016/S0092-8674(01)00518-9 11595178

[B52] Semenza G. L. (2007). Hypoxia-inducible factor 1 (HIF-1) pathway. *Sci. STKE* 2007:cm8. 10.1126/stke.4072007cm8 17925579

[B53] Semenza G. L. (2012). Hypoxia-inducible factors in physiology and medicine. *Cell* 148 399–408. 10.1016/j.cell.2012.01.021 22304911 PMC3437543

[B54] Semenza G. L. (2013). HIF-1 mediates metabolic responses to intratumoral hypoxia and oncogenic mutations. *J. Clin. Invest.* 123 3664–3671. 10.1172/jci67230 23999440 PMC3754249

[B55] Shingo T. Sorokan S. T. Shimazaki T. Weiss S. (2001). Erythropoietin regulates the in vitro and in vivo production of neuronal progenitors by mammalian forebrain neural stem cells. *J. Neurosci.* 21 9733–9743. 10.1523/JNEUROSCI.21-24-09733.2001 11739582 PMC6763035

[B56] Simon M. C. Keith B. (2008). The role of oxygen availability in embryonic development and stem cell function. *Nat. Rev. Mol. Cell Biol.* 9 285–296. 10.1038/nrm2354 18285802 PMC2876333

[B57] Solaini G. Baracca A. Lenaz G. Sgarbi G. (2010). Hypoxia and mitochondrial oxidative metabolism. *Biochim. Biophys. Acta* 1797 1171–1177. 10.1016/j.bbabio.2010.02.011 20153717

[B58] Sorrells S. F. Paredes M. F. Cebrian-Silla A. Sandoval K. Qi D. Kelley K. W. (2018). Human hippocampal neurogenesis drops sharply in children to undetectable levels in adults. *Nature* 555 377–381. 10.1038/nature25975 29513649 PMC6179355

[B59] Sung S. M. Jung D. S. Kwon C. H. Park J. Y. Kang S. K. Kim Y. K. (2007). Hypoxia/reoxygenation stimulates proliferation through PKC-dependent activation of ERK and Akt in mouse neural progenitor cells. *Neurochem. Res.* 32 1932–1939. 10.1007/s11064-007-9390-1 17562163

[B60] Takubo K. Goda N. Yamada W. Iriuchishima H. Ikeda E. Kubota Y. (2010). Regulation of the HIF-1alpha level is essential for hematopoietic stem cells. *Cell Stem Cell* 7 391–402. 10.1016/j.stem.2010.06.020 20804974

[B61] Tambuwala M. M. Cummins E. P. Lenihan C. R. Kiss J. Stauch M. Scholz C. C. (2010). Loss of prolyl hydroxylase-1 protects against colitis through reduced epithelial cell apoptosis and increased barrier function. *Gastroenterology* 139 2093–2101. 10.1053/j.gastro.2010.06.068 20600011

[B62] Tian H. McKnight S. L. Russell D. W. (1997). Endothelial PAS domain protein 1 (EPAS1), a transcription factor selectively expressed in endothelial cells. *Genes Dev.* 11 72–82. 10.1101/gad.11.1.72 9000051

[B63] Wakai T. Narasimhan P. Sakata H. Wang E. Yoshioka H. Kinouchi H. (2016). Hypoxic preconditioning enhances neural stem cell transplantation therapy after intracerebral hemorrhage in mice. *J. Cereb. Blood Flow Metab.* 36 2134–2145. 10.1177/0271678x15613798 26661220 PMC5363661

[B64] Wong B. W. Kuchnio A. Bruning U. Carmeliet P. (2013). Emerging novel functions of the oxygen-sensing prolyl hydroxylase domain enzymes. *Trends Biochem. Sci.* 38 3–11. 10.1016/j.tibs.2012.10.004 23200187

[B65] Yoon D. Pastore Y. D. Divoky V. Liu E. Mlodnicka A. E. Rainey K. (2006). Hypoxia-inducible factor-1 deficiency results in dysregulated erythropoiesis signaling and iron homeostasis in mouse development. *J. Biol. Chem.* 281 25703–25711. 10.1074/jbc.M602329200 16787915

[B66] Yu F. White S. B. Zhao Q. Lee F. S. (2001). HIF-1alpha binding to VHL is regulated by stimulus-sensitive proline hydroxylation. *Proc. Natl. Acad. Sci. U.S.A.* 98 9630–9635. 10.1073/pnas.181341498 11504942 PMC55503

[B67] Zhang C. S. Liu Q. Li M. Lin S. Y. Peng Y. Wu D. (2015). RHOBTB3 promotes proteasomal degradation of HIFalpha through facilitating hydroxylation and suppresses the Warburg effect. *Cell Res.* 25 1025–1042. 10.1038/cr.2015.90 26215701 PMC4559813

[B68] Zhang H. Gao P. Fukuda R. Kumar G. Krishnamachary B. Zeller K. I. (2007). HIF-1 inhibits mitochondrial biogenesis and cellular respiration in VHL-deficient renal cell carcinoma by repression of C-MYC activity. *Cancer Cell* 11 407–420. 10.1016/j.ccr.2007.04.001 17482131

[B69] Zhang J. Guan J. Qi X. Ding H. Yuan H. Xie Z. (2016). Dimethyloxaloylglycine promotes the angiogenic activity of mesenchymal stem cells derived from iPSCs via activation of the PI3K/Akt pathway for bone regeneration. *Int. J. Biol. Sci.* 12 639–652. 10.7150/ijbs.14025 27194942 PMC4870708

[B70] Zhang K. Zhao T. Huang X. Wu L. Y. Wu K. Zhu L. L. (2014). Notch1 mediates postnatal neurogenesis in hippocampus enhanced by intermittent hypoxia. *Neurobiol. Dis.* 64 66–78. 10.1016/j.nbd.2013.12.010 24368168

[B71] Zhang P. Yao Q. Lu L. Li Y. Chen P. J. Duan C. (2014). Hypoxia-inducible factor 3 is an oxygen-dependent transcription activator and regulates a distinct transcriptional response to hypoxia. *Cell Rep.* 6 1110–1121. 10.1016/j.celrep.2014.02.011 24613356

[B72] Zhao T. Zhang C. P. Liu Z. H. Wu L. Y. Huang X. Wu H. T. (2008). Hypoxia-driven proliferation of embryonic neural stem/progenitor cells–role of hypoxia-inducible transcription factor-1alpha. *FEBS J.* 275 1824–1834. 10.1111/j.1742-4658.2008.06340.x 18341590

[B73] Zhu L. L. Zhao T. Li H. S. Zhao H. Wu L. Y. Ding A. S. (2005). Neurogenesis in the adult rat brain after intermittent hypoxia. *Brain Res.* 1055 1–6. 10.1016/j.brainres.2005.04.075 16098951

